# Electronic-components less fully textile multiple resonant combiners for body-centric near field communication

**DOI:** 10.1038/s41598-021-81246-z

**Published:** 2021-01-25

**Authors:** Baptiste Garnier, Philippe Mariage, François Rault, Cédric Cochrane, Vladan Koncar

**Affiliations:** 1 Ecole Nationale Supérieured des Arts et Industries Textiles, Roubaix, France; 2 CNRS, Centrale Lille, UMR 8520- IEMN, Univ. Polytechnique Hauts-de-France, Univ. Lille, 59000 Lille, France

**Keywords:** Energy harvesting, Electrical and electronic engineering, Materials science, Materials for energy and catalysis

## Abstract

Smart and e-textiles have nowadays an important increasing place in the garment industry. The rise of embedded telecommunications, especially smartphones in our pocket, enables us to provide a power source and a wireless link for smart textiles. The main issue is to develop garments able to receive power from smartphones and communicate with them without flexibility and comfort constraints bound to embedded solid-state electronic components. Consequently, this article aims to develop a fully textile NFC combiner to transfer data and power between a smartphone and sensors without any electronic components. It precisely describes textile NFC multiple combiners composed of textile NFC antennas linked by two-wire transmission lines. Also, theoretical analysis, simulations, and experiments have been conducted to adapt the resonant frequency of such structures to the NFC technology (13.56 MHz). Finally, our article generalizes textile NFC combiner resonant frequency equations for multiple combiners with any number of antennas.

## Introduction

E-textiles are part of a larger package commonly known as "smart textiles". E-textiles can be defined by a textile structure (fiber, thread, fabric, or finished product) permanently integrating electrical and/or electronic functionalities. Next, we can also distinguish wearable objects, which are fully functional finished electronic products specially designed to be worn on the body. Finally, by combining these two objects, we can define textile-based end products permanently integrating electrical and/or electronic functionalities designed to be worn on the body, with or without detachable elements.

These different e-textiles find their applications in several fairly distinct fields. Firstly, they can be used in the medical field, for example, by monitoring physiological functions^1,2^. They are also found in personal protective equipment because they can be used to create fabrics with electromagnetic barrier properties^3,4^, or to measure environmental parameters to warn the operator in the event of danger, for example, firefighter clothing^5^. Then, e-textiles can be used in sports to monitor performances and the physiological parameters^6,7^, and also help the recovery^8,9^. The fashion world has also started to take over e-textiles. Indeed, they allow going further in the creations, in particular by adding light^10^ or sound^11^ elements. Finally, the military also uses these materials to improve its camouflage, communication, and protection capabilities^12,13^.

Recent researches have highlighted the development possibilities of wearable sensor networks in textile materials. Lulu Xu et al*.*^14^ have developed a textile NFC coil, handling small fabric deformations, that is combined with an adapted solid-state capacitor to create an antenna resonating at 13.56 MHz. However, the addition of a classic capacitor makes the antenna none fully textile and requires few connection points. As the soldering is always an issue on flexible structures, this may affect the reliability of the system.

Garnier et al*.*^15^ have recently published a paper regarding the prototyping process of a 100% textile NFC antenna, using the embroidered geometrical pattern to create resistance, inductance, and capacity without using any solid-state electronic component. This study provides experimental protocols and results concerning the evaluation of antennas' electric characteristics as well. Also, an NFC relay structure is broached, but the principle has not been generalized and the mechanisms have not been explained in detail. This study established the founding definitions concerning the electrical and geometrical parameters of the textile embroidered antenna, which are the basic elements of the current article.

Finally, Rongzhou Lin et al.^16^ have developed wearable wireless battery-free body sensor networks using near field communication (NFC) to transfer data and power between a smartphone and sensors. The embroidery process has been used to create NFC adapted coils, composed of a conductive thread, on a textile substrate. This structure can be considered as an NFC relay (combiners) enabling to centralize the power supply and the storage device in one coil to deliver them to the other coils, which are paired up with sensors. The conducted study highlighted the possibility to monitor physiological parameters, such as the temperature or the strain, at rest or during the activity. This is made possible by a power adaptation of the different system components (relay/combiner, source, and sensor node) very accurate. The three previous prototypes are quite different in their construction, the first^14^ includes an additional capacity to reach a resonant frequency of 13.56 MHz, consequently, it is not fully textile. The second^15^ focuses on the adaptation of intrinsic parameters to reach the resonant frequency without a precise characterization of energy and data transfer. Finally, the third prototype^16^ deals with the energy transfer efficiency and does not broach the resonant frequency. The main differences are summarized in Table 1.

**Table 1:** Main differences among the recently developed embroidered NFC textile antennae.

	Lulu Xu et al.^14^	Garnier et al.^15^	Rongzhou Lin et al.^16^
Electronic-components	Yes, capacitor	No	No
Quality factor	40 < Q < 50	40 < Q < 50	Undefined
Resonant frequency (MHz)	13,56	13,83	Undefined

However, the absence of capacitors in the textile relay (combiner) electric diagram in Rongzhou Lin et al. article shows that its intrinsic capacity has been neglected. Therefore, the proposed relay has not been optimized for the specific resonating frequency. Nevertheless, when a smartphone or a tag is put in the proximity of one coil, the whole circuit works as an over-coupled system. This means that, as the relay capacity is very small, the resonating frequency is split into two frequencies. The lower frequency depends on the coupling coefficient (*k*) between the smartphone and the coil. This coefficient *k* depends on the distance between the coil and the smartphone and it may be supposed that as the quality factor (*Q)* of the relay is low enough, at a reasonable distance the lower resonating frequency approaches 13.56 MHz. The distance between two resonating frequencies (lower and higher) is equal to $\frac{{\sqrt {k^{2} *Q^{2} - 1} }}{Q}*f_{0}$. For instance, if k*Q = 2 and *Q* = 10 the gap between the two frequencies is equal to $\frac{\sqrt 3 }{{10}}f_{0}$. Therefore, the relay presented in this article is not a resonating one at 13.56 MHz. It just transmits the energy of the signal emitted by the smartphone at NFC frequency. It means that the gain of the relay is not optimized at 13.56 MHz. The advantage of this approach is that it works at any frequency around the NFC standard, but its efficiency is not optimal, as there is no proper resonating frequency. Because of that, the system published in this article is limited in terms of several interconnected antennas. Moreover, the transferred power through the relay is directly related to the linear resistance of the current conductive threads line forming the whole structure. It exists on the market better conductive yarns than those used, with linear resistance values down to approximately 4 Ω.m^−1^^17^. In our previous paper^15^, this low resistance conductive yarn has been used 3 times overlapped to decrease even more the linear electrical resistance down to 1.4 Ω.m^–1^. This allows us to increase the quality factor value, which has results in the power transfer efficiency increase, but also in the decrease of the operating bandwidth narrowed at 13.56 MHz.

In this article, fully textile multiple resonant combiners without any added electronic part such as a capacitor or an inductor, able to resonate without added transmitter or receiver, at the NFC frequency (13.56 MHz) has been developed and fabricated by a genuine textile industrial process. Our combiners are suitable for body-centric NFC communication with a possibility to use any NFC market available compatible smartphone or tag as a transmitter or receiver. Furthermore, a theoretical and simulation analysis of a textile NFC combiner has been conducted to determine the power transfer efficiency when the resonant frequency is adapted. Besides, such a network must be adjustable to a garment; however, its shape has a strong impact on its electrical parameters. Consequently, this paper also provides a method to determine the characteristics of the textile NFC combiners according to their use.

First, a theoretical analysis has been conducted to understand the physical principle of the textile NFC combiners impedance, when they are exposed to an external magnetic field. Then, the same analysis has been realized when the combiner is connected to an impedance analyzer, to compare theoretical and experimental results. Also, this analysis is needed to feed the simulations, which are introduced in the second part. Finally, an experimental approach has been conducted, according to previous analysis and simulations, to evaluate textile NFC combiner geometrical characteristics.

## Results

### Theoretical analysis

**Figure 1:**
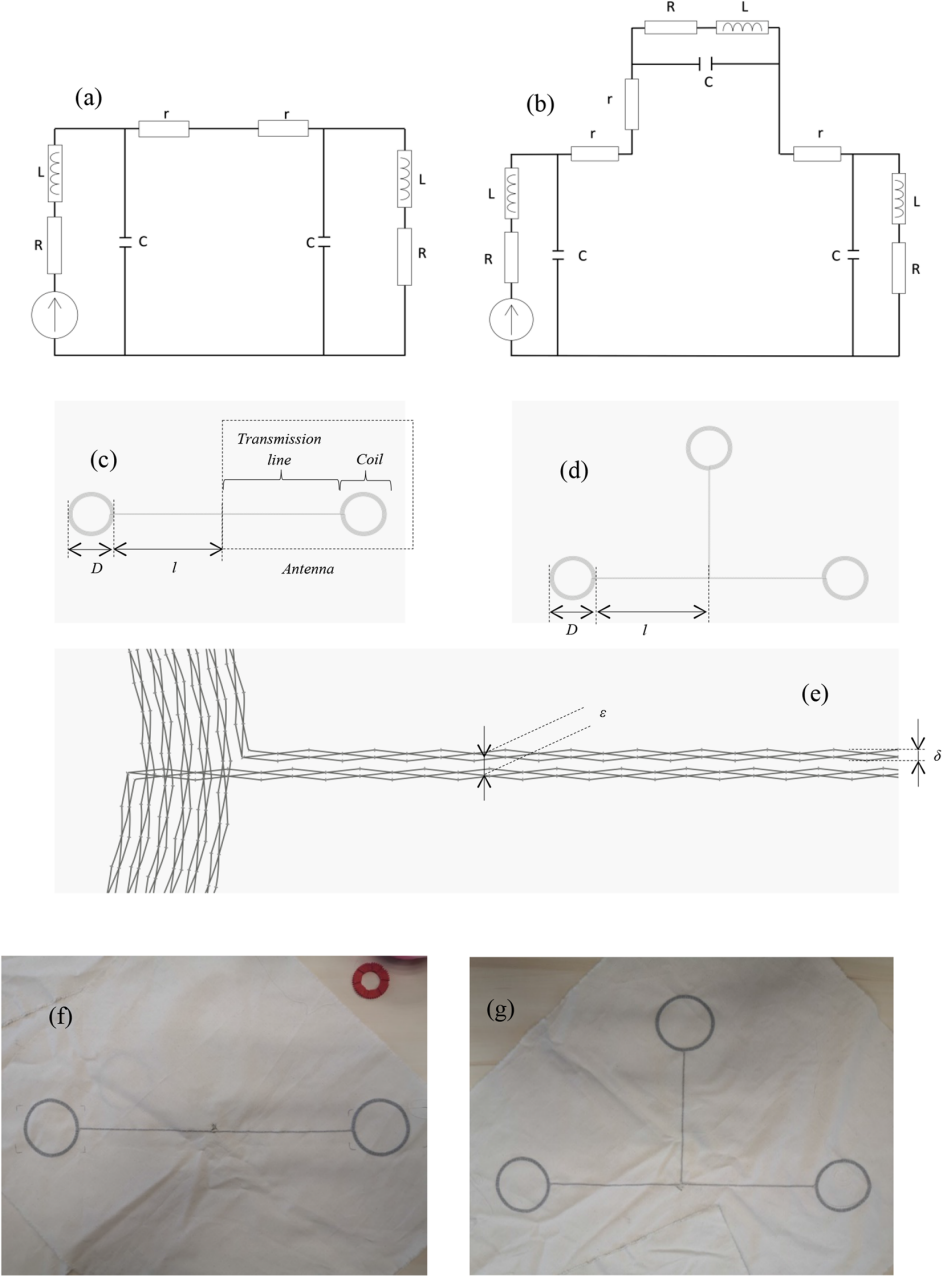
A two and three-antenna combiner electric diagrams (**a**,**b**), and the geometry of the structures including two-antenna and three-antenna, respectively (**c**,**d**), and a schematic of a zoom on the structure (**e**). Where D = 80 mm is the outer diameter of the coil made of 6 turns each, l = 200 mm is the length of the transmission line, δ = 0.5 mm is the width of the current line, and ε = 0.75 mm is the width between two current lines. A two-antenna (**f**) and a three-antenna (**g**) textile combiners prototypes photos.

The textile NFC multiple combiners are assembled RLC circuits; consequently, it is possible to calculate their impedance to predict their resonant frequencies. Textile NFC multiple combiners can be considered as extension cables able to carry a magnetic field (power and data) around the human body. To fit the garment industry requirements related to wearability, clothing comfort, etc., textile NFC multiple combiners should be entirely made of textile materials. The combiner structure is made of twisted polyester/copper highly flexible and processable conductive yarn embroidered on cotton fabric as a substrate. This substrate fabric can be made of different structures (woven, knitted, non-woven, etc.) and materials (polyester, acrylic, wool…). The textile resonating combiners may include several plane circular coil antennas bounded by a two-wire transmission line. Moreover, the 3 overlapped textile conductive threads enable to decrease of the current line linear resistance down to 1.4 Ω.m^−1^, which is the optimal compromise among the cost, efficiency, processability, and comfort. The associated electrical diagram is shown in Fig. 1. (a) for a two-antenna combiner and Fig. 1. (b) for a three-antenna combiner, while (c) and (d) represent the geometry schematics of the actual structures including two-antenna and three-antenna, respectively. An induced voltage source has been drawn on the electrical diagram to take into account a magnetic coupling of an external transmitter (non-presented) with one of the coils of the combiners. Figure 1e–g show the detail of the conductive line composed of three overlapped embroidered bobbin yarns, and 2 and 3 combiner prototypes respectively.

Multiple combiners are resonated when the total impedance (Z_n coils_; where n: number of coils) seen by the source is purely real to avoid to stock the energy in reactive components. To determine the responses of such structures when they are stimulated by an induced magnetic field source, the expression of the resonant frequency has been calculated. The theoretical expression of the impedance of two and three-antenna combiners is displayed by Eq. (1) and (3), where $R$ (Ω) is the resistance, $L$ (Henry) is the inductance, $C$ (Farad) is the capacity, $f_{0}$ (Hertz) is the resonant frequency, ω (rad/sec), *Z* (Ω) is the impedance, and *j* the complex number. To simplify the mathematical expressions, the small resistive part of each antenna noted “*r*” shown in Fig. 1 has been neglected according to the values observed later in the measurement part of the study. The theoretical study highlights that the resonant frequency has still the same expression regardless of the number of antennas forming the combiner.

It can be shown that the condition for canceling the imaginary part of that expression at the resonant frequency is given by Eq. (2) for 2 coils combiner:

1 $$Z_{2 - coil} = R + jL\omega + \frac{{R\left( {1 - 2LC\omega^{2} } \right) + 2RLC\omega^{2} + j\left[ {L\omega \left( {1 - 2LC\omega^{2} } \right) - 2R^{2} C\omega } \right]}}{{\left( {1 - 2LC\omega^{2} } \right)^{2} + 4R^{2} C^{2} \omega^{2} }}$$

2 $$\begin{aligned} & \omega _{{0,2 - coil}}^{2} = \frac{1}{{LC}}\left( {1 - \frac{1}{{2Q^{2} }}} \right) \approx \frac{1}{{LC}}if\;Q = \frac{1}{R}\sqrt {\frac{L}{C}} \gg 1 \\ & \quad or\;f_{{0,2 - coil}} = \frac{1}{{2\pi \sqrt {LC} }} \\ \end{aligned}$$

3 $$Z_{3 - coil} = R + jL\omega + \frac{1}{{jC\omega + \frac{1}{{2\frac{R + jL\omega }{{1 + jRC\omega - LC\omega^{2} }}}}}}$$

It can be shown that the condition for canceling the imaginary part of that expression at the resonant frequency is given by Eq. (4) for 3 coils combiner:

4 $$\begin{aligned} & \omega _{{0,3 - coil}}^{2} = \frac{1}{{LC}}\left( {1 - \frac{1}{{2Q^{2} }}} \right) \approx \frac{1}{{LC}}~~if~~Q = \frac{1}{R}\sqrt {\frac{L}{C}} \gg 1 \\ & \quad \quad \quad \quad or~~f_{{0,~3 - coil}} = \frac{1}{{2\pi \sqrt {LC} }} \\ \end{aligned}$$

An SMA connector has been inserted in the circuit to evaluate experimentally the impedance of the combiner. The theoretical impedance calculation highlights that the relay resonant frequency is impacted by the addition of an SMA connector, which is assimilated to a 3 pF capacity (noted *C*_*SMA*_). Equation 5 displays the results of mathematical development.

In this configuration, both impedances are connected in series to the SMA connector capacity. Consequently, the total impedance ($Z_{tot} = 2.Z$) can be expressed by the following Eqs. (5) and (6).

5 $$Z_{tot 2 - coil} = \frac{2R + j2L\omega }{{1 + j2R\left( {\frac{C}{2} + C_{SMA} } \right)\omega - 2L\left( {\frac{C}{2} + C_{SMA} } \right)\omega^{2} }} + 2r$$

Hence, the resonant frequency can be expressed by:

6 $$f_{0} = \frac{1}{2\pi }\sqrt {\frac{1}{{LC^{\prime } }} - \frac{R}{{L}}} \approx \frac{1}{{2\pi \sqrt {LC^{\prime } } }} \quad if\;R \ll \sqrt {\frac{L}{{C^{\prime } }}} \;and\;C^{\prime } = C + 2C_{SMA}$$

The same reasoning can be applied to a three-antenna combiner and generalized for an N-antenna combiner:

7 $$Z_{tot 3 - coil} = \frac{3R + j3L\omega }{{1 + j3R\left( {\frac{C}{3} + C_{SMA} } \right)\omega - 3L\left( {\frac{C}{3} + C_{SMA} } \right)\omega^{2} }} + 3r$$

8 $$Z_{tot N - coil} = \frac{NR + jNL\omega }{{1 + jNR\left( {\frac{C}{N} + C_{SMA} } \right)\omega - NL\left( {\frac{C}{N} + C_{SMA} } \right)\omega^{2} }} + Nr$$

Hence, the resonant frequency can be expressed by:

9 $$f_{0} = \frac{1}{2\pi }\sqrt {\frac{1}{{LC^{\prime } }} - \frac{R}{{L}}} \approx \frac{1}{{2\pi \sqrt {LC^{\prime } } }} \quad if\;R \ll \sqrt {\frac{L}{{C^{\prime } }}} \;and\;C^{\prime } = C + NC_{SMA}$$

The introduction of the SMA connector has the effect of reducing the resonance frequency of the 2-antenna combiner compared to a single antenna. The study of this particular case confirms the theoretical and the simulation results regarding the further experimental measurements. The new expression of the combiner's total capacity makes it possible to apply a corrective value to the resonant frequency value, measured by an impedance analyzer.

Another parameter affecting the power transfer efficiency is the quality factor (*Q*) of the antennas forming the NFC relay. It can be defined as the ratio between the resonant frequency value, $f_{0}$ and the width of the bandwidth $\Delta f$. It can also be defined as the ratio of the inductance and pulsation product on the resistance, displayed in Eq. (10).

10 $$Q = \frac{{f_{0} }}{{\Delta f{ }}} \;\;or\;\;Q = \frac{L \cdot \omega }{R}$$

The quality factor is supposed to evaluate the system quality and the amount of power transferred through the NFC relay structures. The circuit resistance is fundamental in the power transfer efficiency, but the resistive part of the circuit is brought only by the conductive textile yarn. Consequently, it is necessary to control the linear resistance of the circuit to improve the amount of power transferred (high value of *Q*) or to increase the bandwidth (low value of *Q*). The conductive thread choice becomes decisive in the design. Several types of textile conductive yarns are already present in the market; however, the most conductive yarns include metallic filament, such as copper. The thread used in this study is the Datatrans from “Tibtech”, France, and its linear resistance is around 4 Ω.m^−1^. To control further the current line linear resistance, the conductive yarn can be overlapped several times to adjust the total resistance value to the desired quality factor.

### Simulation

**Figure 2:**
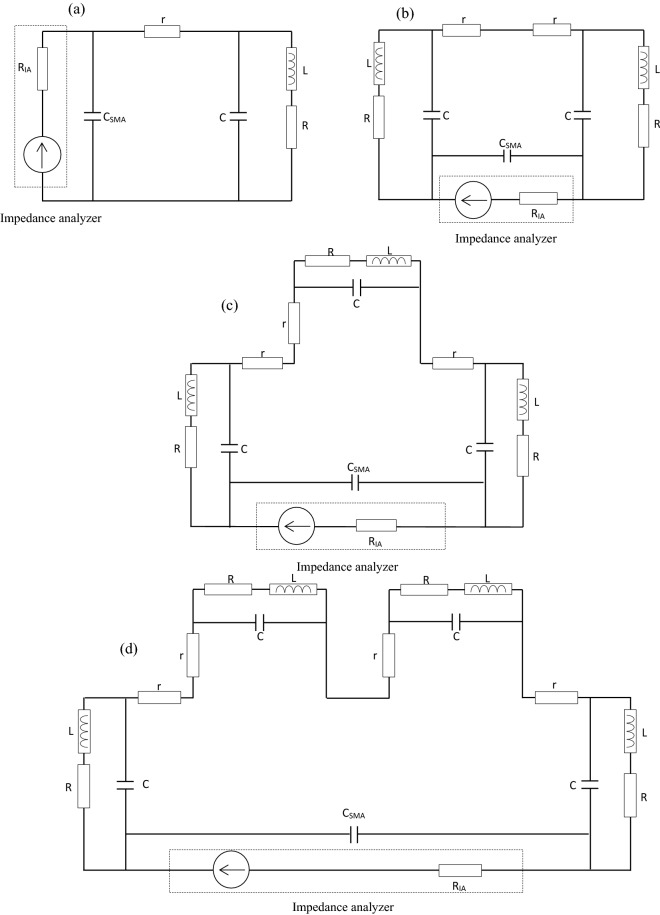
Electric diagrams used in LTspice simulation for NFC combiner composed of (**a**) one, (**b**) two, (**c**) three, and (**d**) four-antenna.

**Figure 3:**
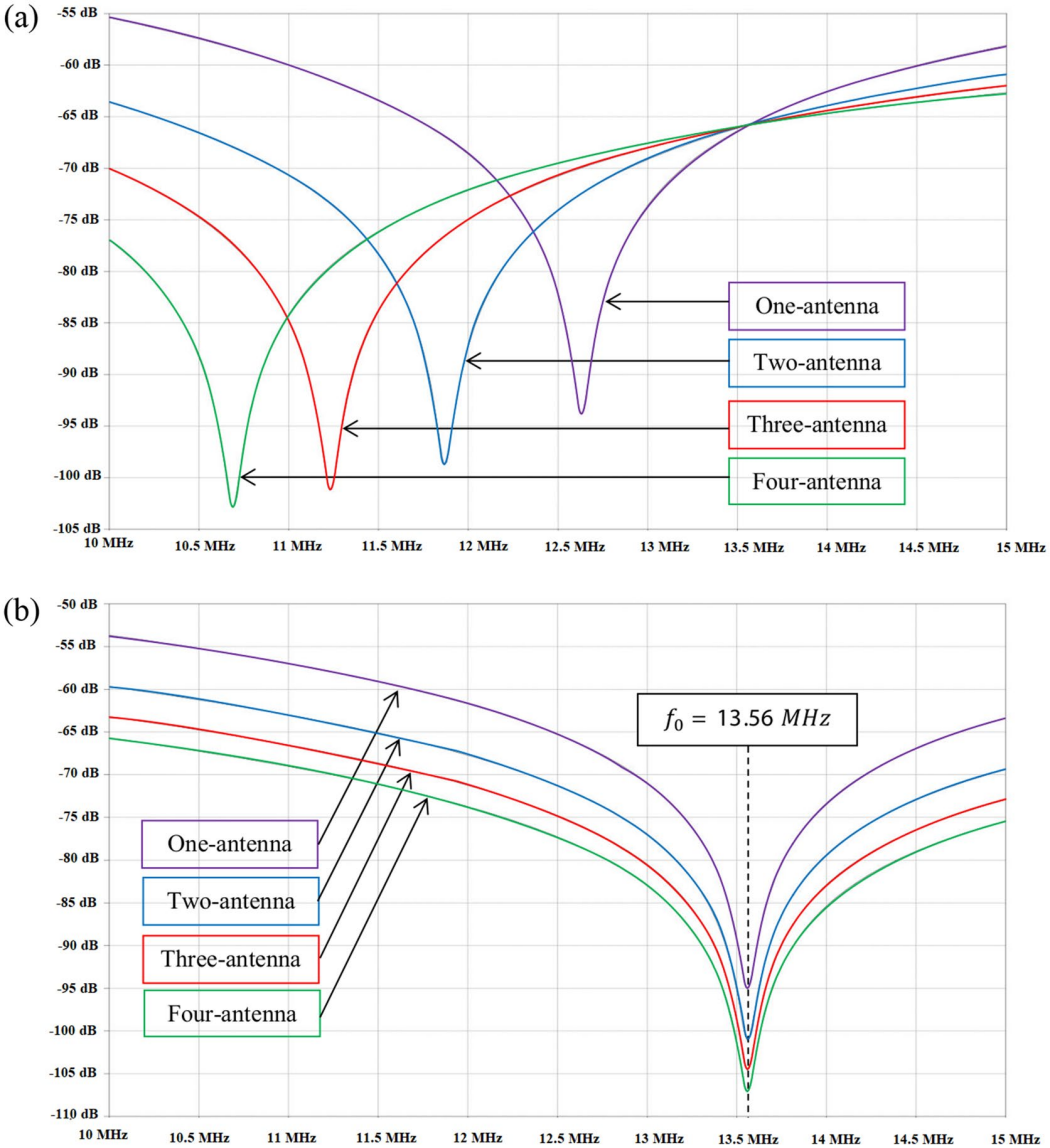
Simulated current in the branch of the impedance analyzer in dBA of the textile NFC combiners versus the frequency. (**a**) Current without taking into account *C*_*SMA*_. (**b**) Current with taking into account *C*_*SMA*_ (*C’* = *C* − *N.C*_*SMA*_).

To confirm the previous theoretical projection, several digital simulations have been conducted. First, the electrical responses of the combiner have been simulated by LTspice (Linear Technology Corporation) software. Combiners composed of one to four-antenna have been simulated and their electrical diagrams used for the simulation are displayed in Fig. 2a–d. The structure's responses are shown in Fig. 3a,b. Simulated voltage injection is made symmetrically to model the presence of the RLC meter connected via an SMA connector and its internal resistance *R*_*IA*_. The values of the components are derived from the experiments.

**Figure 4:**
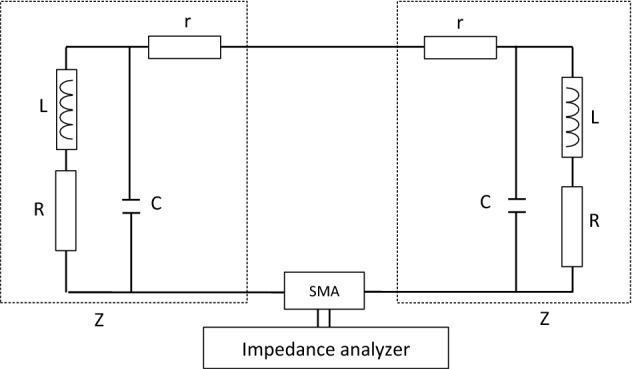
Electric diagram of the textile NFC combiner connected to an impedance analyzer.

These simulations aim to evaluate the combiners' responses to a magnetic field stimulus but also to confirm the theoretical model when compared to the experimental results. The electric diagrams presented in Fig. 4 contain an additional capacity brought by the SMA connector, which is around 3 pF. The simulation results are presented in Fig. 3. The graph displays the evolution of the current in the branch of the impedance analyzer in dBA versus the frequency, where the purple line is the four-antenna combiner, the green line is the three-antenna combiner, the blue line is the two-antenna combiner and the red line is the one-antenna structure. The simulations highlight that the number of antennas decreases the resonant frequency and the efficiency of the combiner.

The presence of an SMA connector into the textile NFC combiner leads to a linear decrease of the resonant frequency (regarding Eq. (11)), depicted by a minimum peak in the resistance of each coil, at the resonant frequency, shown in Fig. 3a. Indeed, the graphs magenta, blue, red, and green, respectively correspond to the one, two, three, and four-antenna combiners. Then, the previous theoretical study on the textile NFC combiner connected to an impedance analyzer gives a corrective value for the circuit's total capacity. If the presence of the capacity of an SMA connector is taken into account then the tuning capacity of the loop has to be decreased by the value of *C*_*SMA*_, i.e. *C’* = *C − N.C*_*SMA*_ to measure the right resonant frequency with the RLC meter (minimum current means that the impedance is maximal and that the textile combiner is resonating). Consequently, Fig. 3b shows the responses of the current in the branch of the impedance analyzer in dBA for all the structures with the corrective value applied (with the same color code). The resonant frequencies are all centered on 13.56 MHz and a small attenuation is highlighted when the number of antennas increases, provoked by the increase of the two wires transmission lines' electrical resistance.

The values used in the simulation and presented in Table 2 were chosen according to a previous study on the textile NFC antennas^15^.

**Table 2:** Simulation values.

*R*	*L*	*C*	*C*_*SMA*_	*r*	*f*_0_	*Q*
3.12 Ω	7 µH	13.68 pF	3 pF	0.3 Ω	13.56 MHz	191

The number of antenna in the structure modifies the resonant frequency, but the study predicts the resonant frequency shift and enables us to compensate its effect by influencing the intrinsic capacity of each antenna. For example, the distance between two current lines or the length of the transmission line modifies the intrinsic antenna capacity.

Also, body movements will modify the 3D geometry of the structure. However, the total length of the current line will be the same, so the resistance. The intrinsic capacity depends on the length of the current line and the distance between two current lines and these parameters will be unchanged by the deformation. Finally, the inductance is the only characteristic which can be modified by the body movement, if the deformation takes place on the coils. But, the resonant frequency modification should remain very low, and the value of the quality factor (~ 40–50) enables us to compensate for this shift.

**Figure 5:**
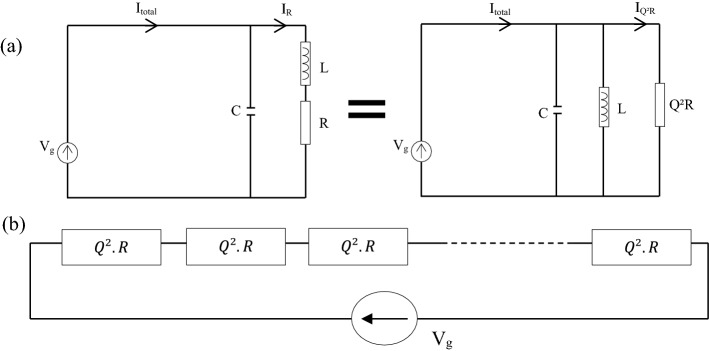
Simplified electric diagram of N-coil combiner at the resonance frequency.

In the case of a single-coil, the one-antenna electric diagram can be replaced by a parallel electric diagram, displayed in Fig. 5a, i.e. that the series resistance *R* becomes a parallel resistance *R*_*p*_ where its value is recalculated to $Rp = Q^{2} \cdot R$, assuming $Q \gg 1$. Consequently, the current in the circuit can be expressed by following Eq. (11).

11 $$I_{{Q^{2} R}} = \frac{{V_{g} }}{{Q^{2} R}}$$

At the resonance, the total N coils combiner circuit can be replaced by the one presented in Fig. 5. The current circulated in the simplified loop can be expressed with Eq. (11). For instance, a 6 dB gap appears between the current in the 2 coils combiners (blue graph, Fig. 3a) and the 4 coil combiner (green graph, Fig. 3a). Figure 5a shows the transition between the real electric diagram and its equivalent parallel electric circuit, with *V*_*g*_ is the ideal voltage source. Figure 5b displays a simplified equivalent electric diagram for an *N*-antenna textile NFC combiner, where the antenna is placed in parallel. This configuration simplifies the current calculation, expressed by Eqs. (12) and (13).

12 $$I_{loop} = \frac{{V_{g} }}{NQR}$$

13 $$20\log_{10} \left( {\frac{{I_{loop, Ncoils} }}{{I_{{loop, \left( {N + 1} \right)coils}} }}} \right) = 20\log_{10} \left( {1 + 1/N} \right)$$

**Figure 6:**
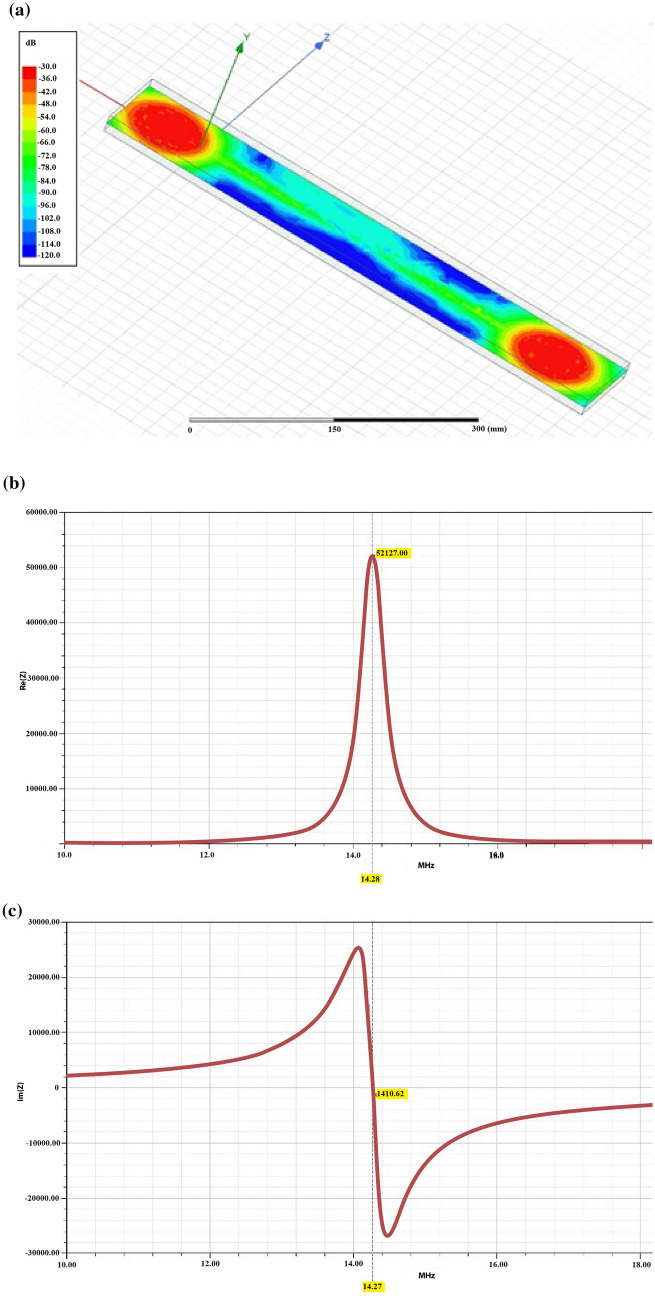
2D cartography of the magnetic field amplitude provided by a 2-coil combiner at 13.56 MHz (**a**) real (**b**) and imaginary (**c**) part of the impedance of a 6-turn coil connected to a 200 mm long two wires rectilinear transmission line.

A second simulation method, using Ansys HFSS software, has been used to characterize the textile NFC combiner transmitted magnetic field. It allows finding the diameter of a conducting wire equivalent to the actual yarn provided by Tibtech. This kind of yarn is built with 4-strands 3 wires twisted with two different pitches values. Thus, it looks like a special Litz wire. Conversely, the equivalent conductor is made of a single wire with a circular cross-section that gives a similar behavior as the real yarn. For this 2 coils combiner stimulated by a 13.56 MHz magnetic field source, the magnitude of the magnetic field along the y-axis (normal to the plane of the whole geometrical structure) has been computed and displayed in Fig. 6a. Figure 6b,c show also the values of the real and imaginary part of the impedance of a one only 6-turn coil connected to a 200 mm long two-wire rectilinear transmission line. It is to be noted that the defined equivalent conductor provided a resonant frequency slightly greater than 13.56 MHz, namely 14.3 MHz that exhibits around 5% error.

### Prototyping

To make the antennas, and therefore the combiner, resonate at the right frequency, it is necessary to bring a resistive, an inductive, and a capacitive element to the circuit. The textile conductive yarn constitutes the resistive part as it possesses non-negligible linear resistance (1.4 Ω.m^−1^). This resistance is evenly distributed along the current line. But the distributed resistances are in series, which enables them to consider as an equivalent resistance thanks to the additional series resistance law.

**Figure 7:**
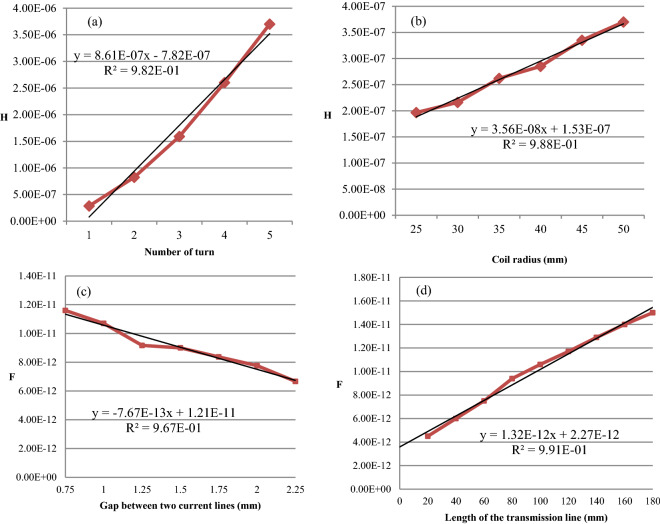
Evolution of the coil inductance versus (**a**) the number of turns and (**b**) the radius^18^. (**c**) Evolution of the intrinsic capacity of a textile transmission line (*l* = 100 mm) versus the gap between its two current lines and (**d**) the evolution of the intrinsic capacity of a textile transmission line (*d* = 0.75 mm) versus its length.

The inductance part is brought by the planar spiral geometry of the current line. According to the impedance measurements conducted on a different type of planar spiral antennas. An experimental model to determine the antennas geometry versus the using constraint has been realized and expressed in Eqs. (14). Figure 7 shows the evolution of the inductance versus (a) the number of turns and (b) the radius

14 $$\begin{aligned} & L = \left( {0.816*Nb - 7.82} \right)10^{ - 7 } H, \quad if\,r = 40\;{\text{mm}} \\ & L = \left( {0.356*r + 1.53} \right)10^{ - 7} H, \quad if\;Nb = 1 \;{\text{turn}} \\ \end{aligned}$$

where *Nb* is the number of turns and *r* (mm) is the radius of the coil.

The embroidery process has been used to develop a planar spiral antenna. It enables to design of the structure with precise dimensions, essential for matching antennas parameters to the source. Moreover, the conductive yarns have been used as a bobbin yarn to avoid too much mechanical stress and consequently deterioration. In the embroidery process, there are two yarns, the sewing yarn going through the needle eye, and the bobbin yarn that is placed under the fabric. Mechanical stresses are much higher for the sewing yarn^15^. Also, the use of the bobbin makes the conductive yarn to be dropped off in a straight line, unlike the sewing yarn which goes through the substrate.

The very short distance between two current lines and the superposition of three insulated conductive yarns involve the creation of a capacitive effect. The developed antennas have necessary an intrinsic capacity. The influence of the length and the gap of the transmission line on the intrinsic capacities are displayed in Fig. 7c,d, and an approximation can be expressed by Eq. (15), which enables to predict the total capacity of an antenna.

15 $$\begin{aligned} & C = \left( { - 7.67*d + 121} \right)10^{ - 13} F, \quad if\,l = 100\;{\text{mm}} \\ & C = \left( {1.32*l + 2.27} \right)10^{ - 12} F, \quad if\;d = 0.75\;{\text{mm}} \\ \end{aligned}$$

where *d* (mm) is the distance between two current lines and *l (*mm*)* the length of the current line.

The conductive yarn used to create the antennas is insulated by polyester filaments to avoid the electric connection between the multiple yarns and bring a capacitive effect to the combiner. The very thin gap between current lines, around 0.75 mm, also gives a more capacitive effect on the structure. Consequently, the combiner capacity is influenced by the length of the current line and the gap between them.

Then, an experiment on a textile NFC combiner connected to an impedance analyzer has been conducted to confront simulation and experimental results. A 6-turn coil antenna with a 400 mm total length (2*l) of the transmission line has been connected to an Agilent 4964A via an SMA connector, to perform the measurement; the results are shown in Table 3.

**Table 3:** The resonant frequency of the textile NFC structure according to the number of coils.

Number of coils	1	2	3
Resonant frequency (MHz)	15.86	11.99	9.83

As expected from the simulation results, the resonant frequency is shifted when the number of antennas increases. The resonant frequency value is 15.86 MHz for a single-antenna structure, then 11.99 MHz for a two-antenna combiner, and 9.83 MHz for a three-antenna combiner. This experimental result enables to confirm the previous simulation, even if the values of simulation and experimental do not match perfectly, probably due to the soldering issues of an SMA connector implying different capacities. However, the evolution tendency is the same in both cases.

## Discussion

Through this study, we have demonstrated how to adapt textile NFC antenna and combiner in terms of resonant frequency to optimize the data and power transfer. Moreover, the established equations and results allow the development of several types of textile NFC combiners, according to their use. Rongzhou Lin et al*.* article concerned the feasibility to use a textile NFC relay to monitor body sensors without a battery, by focusing on the feasibility of the power and data transfer. This new article completes it by focusing on the resonant frequency conditions to improve the power and data transfer capacity.

A complete study of the resonant frequency physic phenomenon in a textile NFC combiner ensures control of the structure quality factor and consequently the bandwidth. Indeed, garments are dedicated to enduring abundant types of stress, such as abrasion, stretching, dirt, and washing. All these degradations can lead to modifications of textile NFC combiners' electrical parameters and make the structure ineffective. Consequently, the anticipation of such phenomena is essential to guarantee lasting efficiency and precise control of the resonant frequency. It enables to adjust the combiner bandwidth to be adapted to its particular use. An increase in bandwidth makes the combiner less sensitive to damages and deformations but also has the consequence of reducing the amount of energy transferred (because the *Q* factor also decreases).

The calculation and the prototyping results have facilitated a procedure to create textile NFC combiners. These structures are consequently usable in different fields, not only the garment. All electrical and geometrical values are interconnected to make a combiner to resonate exactly at 13.56 MHz. The presented prototyping method helps to define the combiner geometry according to the constraints generated by its use.

In terms of other applications, textile NFC multiple combiners can be used to instrument furniture fabric. For example, textile NFC combiners embedded tablecloth could provide an easy way to transfer data between all meeting participants. Then, textile NFC structures with strong power transfer efficiency could power supply sensory network embedded garments. These structures can also be combined with other telecommunication technologies, especially the ISM band communication working at 2.4 GHz. The use of these two communication bands to create hybrid textile dual-band combiners could improve the performances of sensory network embedded garments. Several functioning prototypes have been realized and manufactured the following aforementioned procedures; two of them are shown in Fig. 1f,g.

## Methods

### Textile NFC combiner fabrication

The textile NFC combiners were designed by the computer-aided design software from the ZSK Company, Germany, according to an anterior study (15) on the electrical characteristics of textile antennas. Then, the structures were embroidered by a "JF-0215–495" from the ZSK Company, Germany, on cotton fabric. The upper thread used was a basic hosiery cotton yarn, while the bobbin thread used was a textile electric conductive yarn from the "Tibtech" Company, France, composed of 4 twisted copper filaments on a polyester core and isolated by a filaments PET layer. The current line is composed of 3 overlapped conductive yarns to improve its conductivity.

### Characterization

All the characterizations have been conducted under the conditions of a temperature of 21 °C and relative humidity of 65% of our standardized laboratory. The impedance analyzer used was a calibrated Agilent 4964A. The prototypes were connected to the impedance analyzer and by using standard SMA connectors whose capacity has been measured and added to the calculation.

### Numerical simulation

The first numerical simulations have been realized by using the LTspice electrical simulation software. All the electrical values used in these simulations came from a previous publication focusing on the textile antenna electrical characteristics. The second simulations have been realized by using the Ansys HFSS magnetic field simulation software. A 3D model of the combiners has been created in which the electrical characteristics of the textile conductive thread have been implemented to simulate the emitted magnetic field.
